# c-Src activation promotes nasopharyngeal carcinoma metastasis by inducing the epithelial-mesenchymal transition via PI3K/Akt signaling pathway: a new and promising target for NPC

**DOI:** 10.18632/oncotarget.8634

**Published:** 2016-04-07

**Authors:** Liangru Ke, Yanqun Xiang, Xiang Guo, Jinping Lu, Weixiong Xia, Yahui Yu, Yongjian Peng, Li Wang, Gang Wang, Yanfang Ye, Jing Yang, Hu Liang, Tiebang Kang, Xing Lv

**Affiliations:** ^1^ State Key Laboratory of Oncology in South China, Collaborative Innovation Center for Cancer Medicine, Guangzhou, China; ^2^ Department of Nasopharyngeal Carcinoma, Sun Yat-Sen University Cancer Center, Guangzhou, China; ^3^ Department of Biostatistics and Epidemiology, School of Public Health, Sun Yat-Sen University, Guangzhou, China; ^4^ Medical Research Center and Clinical Laboratory, Zhuhai Hospital, Jinan University, Zhuhai People's Hospital, Zhuhai, China

**Keywords:** nasopharyngeal carcinoma, metastasis, c-Src activation, therapy target, epithelial-mesenchymal transition

## Abstract

Aberrant activation of cellular Src (c-Src), a non-receptor tyrosine kinase, could promote cancer progression through activating its downstream signaling pathways. However, the roles of c-Src and phosphorylated-Src (p-Src) in nasopharyngeal carcinoma (NPC) progression are rarely investigated. Herein, we have identified high c-Src concentrations in the serum of NPC patients with distant metastasis using high-throughput protein microarrays. Levels of c-Src in serum and p-Src in human primary NPC samples were unfavorable independent prognostic factors for cancer-specific survival, disease-free survival, and distant metastasis-free survival. Depletion or inactivation of c-Src in NPC cells using sgRNA with CRISPR/Cas9 system or PP2 decreased cell viability, colony formation, migration and invasion *in vitro* and metastasis *in vivo*. In contrast, these malignancies could be up-regulated by overexpressed c-Src in a NPC cell line with low-metastasis potential. Furthermore, p-Src was involved in promoting NPC cell metastasis by inducing the epithelial-mesenchymal transition (EMT) process via activating the PI3K/Akt pathway and cytoskeleton remodeling. The p-Src-induced EMT process could be retarded by PP2, which mediated by down-regulating the PI3K/Akt pathway. In conclusion, elevated levels of c-Src in serum and p-Src in primary NPC tissue correlated with poor outcomes of NPC patients. And aberrant activation of c-Src facilitated NPC cells with malignant potential, especially metastasis ability, which mediated by the PI3K/Akt pathway activation and sequentially induced the EMT process. These findings unveiled a promising approach for targeted therapy of advanced NPC.

## INTRODUCTION

Nasopharyngeal carcinoma (NPC) is a malignant tumor originating from the epithelium of the nasopharynx. And NPC is endemic in Southern China and Southeast Asia, with an incidence rate varying from 20 to 50 cases per 100,000 people [1–4]. Advances in chemotherapy and radiotherapy have significantly improved patient outcomes [5, 6]. Unfortunately, due to the high potential of metastasis and invasiveness, regional recurrence and distant metastasis remain major causes of failure in advanced NPC [7]. Thus, identifying remarkable biomarkers and understanding the underlying mechanisms of NPC could define molecular phenotypes of patients with NPC, which could help to select high-risk subpopulations of NPC patients for precision therapy.

The non-receptor tyrosine kinase c-Src, one of the nine Src family kinases (SFKs), has been implicated to be involved in fundamental physiological and pathological processes, including carcinogenesis and tumor progression [8, 9]. All SFKs share similar structural features and are composed of a unique NH2-terminal region, four conserved Src homology domains (SH1 to SH4), and a C-terminal regulatory domain [10]. Phosphorylation of tyrosine Y416 in mice (corresponding to human Y419) in the SH1 domain alters the conformation and increases the intrinsic kinase activity of c-Src [11]. In epithelium cells, active c-Src regulates the focal adhesion and adherens junctions, epithelial-mesenchymal transition (EMT) [12], metalloproteinase (MMP) production [13], phosphorylation of β-catenin [14] and translocation of β-catenin from the cytosol to nucleus [15], all of which reduce cell-cell and cell-matrix junctions and provide cells with increased invasion and motility potential. Notably, c-Src is at the cross-roads of several signaling pathways, including Ras/Raf/extracellular signal-regulated kinase (ERK) 1/2, phosphatidylinositol 3-kinase (PI3K)/Akt, and signal transducer and activator of transcription (STAT) 3 [16–18], resulting in cell proliferation, survival, invasion, migration and angiogenesis. Recent studies focused on c-Src have developed several efficient targeted therapeutic agents, including dasatinib, AZD0530, and SKI-606. Among these agents, dasatinib has been extensively verified to improve treatment response in imatinib-resistant chronic myeloid leukemia [19]. Although the role of p-Src in the progression of multiple types of cancer has been extensively verified, the correlation between p-Src and NPC has not yet been sufficiently investigated. Several studies have demonstrated that SFKs are involved in the pathogenesis of head and neck squamous cell carcinomas (HNSCCs). TNFα can induce the expression and activation of some SFKs (c-Src, c-Yes, Fyn, and Lyn) in HNSCC cell lines [20]. The combination STAT3 and SFKs inhibitor dasatinib significantly induced apoptosis and decreased proliferation of HNSCC cells [21]. Although NPC is a type of HNSCC, NPC has unique pathogenesis characteristics and is not included in most of HNSCC studies. Herein, we hypothesized that c-Src activation is an unfavorable prognostic factor for NPC and could be used as a therapeutic target in NPC patients. We therefore evaluated the prognostic value of p-Src in NPC samples and determined *in vitro* and *in vivo* roles of p-Src in NPC cells.

Epithelial-mesenchymal transition (EMT), a fundamental process in embryonic development, has been implicated in cancer progression [22–24]. Several molecules, such as JAM-A and EBV-miR-BART7-3p, have been suggested to regulate EMT via the PI3K/AKT pathway. Particularly, p-Src has been shown to promote the metastasis of pancreatic cancer and HNSCCs by inducing the EMT process [25, 26]. Src-mediated phosphorylation induces the ubiquitination and endocytosis of E-cadherin, which directly affects cell junctions to increase cell motility [27–29] or vascular permeability to permit cancer cells to metastasize via intravasation and extravasation [30, 31]. However, whether p-Src can promote NPC cells to metastasize by inducing the EMT process and its predominant downstream signaling pathways in NPC remains unclear.

In the present study, we evaluated the levels of c-Src and p-Src in clinical samples, including the serum and tumor tissues of patients with NPC, and examined the correlations between p-Src levels and outcomes. We identified that p-Src promoted NPC cell metastasis by inducing the EMT process through activating the PI3K/AKT signaling pathway; particularly, 4-amino-5-(4-chlorophenyl)-7-(t-butyl)pyrazolo[3,4-d]pyrimidine (PP2), an inhibitor of SFKs, reduced metastatic potential by reversing the EMT process *in vitro* and *in vivo* by suppressing PI3K/AKT activation. Collectively, our study highlights the role of p-Src in metastatic promotion and the potential clinical application of c-Src inhibition in NPC.

## RESULTS

### Elevated serum levels of c-Src (sc-Src) and p-Src(Y419) (sp-Src) were correlated with poor outcomes in NPC patients

#### Elevated sc-Src was correlated with poor outcomes of advanced NPC patients

**Figure 1:**
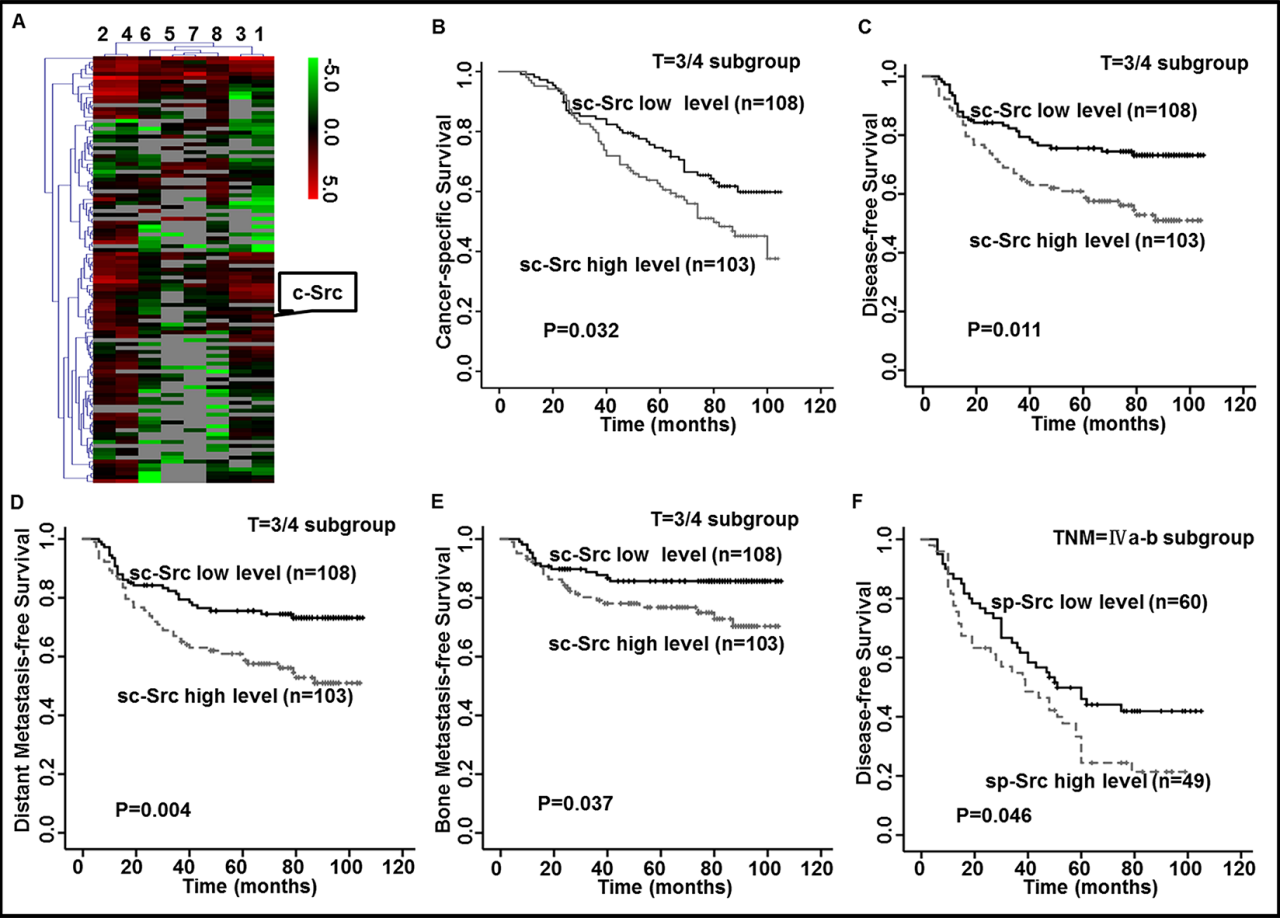
Elevated levels of c-Src (sc-Src) and phospho-Src (Y419) (sp-Src) were correlated with poor outcomes in nasopharyngeal cancer (NPC) patients (**A**) Heatmap showed the serum protein expression profiles of NPC patients with (ID 1–4) or without (ID 5–8) distant metastasis. Red and green indicate the up-regulation and down-regulation, respectively. (**B**–**F**) Kaplan–Meier analysis was used to estimate 9-year cancer-specific survival (CSS), disease-free survival (DFS), distant metastasis-free survival (DMFS) and bone metastasis-free survival (bone-MFS) for NPC patients based on sc-Src or sp-Src. The 9-year CSS (B), DFS (C), DMFS (D) and bone-MFS (E) for the 211 patients in the T = 3/4 subgroup of the ELISA set, based on sc-Src. The 9-year DFS (F) for the 109 patients in the TNM stage = IVa–b subgroup of the ELISA set, based on sp-Src. *P* values were calculated using unadjusted log-rank tests.

The serum protein spectra of NPC patients with distant metastasis were compared to spectra of NPC patients without distant metastasis using high-throughput protein microarrays. This analysis revealed 61 proteins that were elevated and 17 proteins that were lower in patients with distant metastasis. Notably, sc-Src was among the elevated proteins in NPC patients with distant metastasis (*P* = 0.024, Figure 1A).

To determine the relationship between sc-Src levels and NPC patient outcomes, we quantified the concentration of sc-Src in the serum of NPC patients without distant metastasis. The range of sc-Src of these patients varied from 1.12 pg/ml to 31.05 pg/ml, and the cut-off value was 13.24 pg/ml according to ROC curve based on the occurrence of progression in NPC patients. Therefore, patients were classified into high and low groups.

The baseline clinical characteristics, including age, sex, histology type, N stage, clinical stage and treatment, were not significantly different between two groups except for the T stage (*P* = 0.005, Supplementary Table S1). Further Spearman rank correlation analysis demonstrated that high concentrations of sc-Src positively correlated with advanced T stage (correlation coefficient *r* = 0.165, *P* = 0.005, Supplementary Figure S1). Kaplan–Meier survival analyses were performed in subgroups to exclude the bias of imbalance of T stage, expectedly, patients with elevated sc-Src had worse 9-year cancer-specific survival (CSS), disease-free survival (DFS), distant metastasis-free survival (DMFS) and bone metastasis-free survival (bone-MFS) (Figure 1B–1E) compared with sc-Src-low patients in the T = 3/4 subgroup, with no significant difference in the baseline clinical characteristics (Supplementary Table S2).

#### Elevated sp-Src was correlated with poor outcomes of advanced NPC patients

Since c-Src was reported to promote cancer progression through its aberrant phosphorylation activity, the level of sp-Src was also evaluated in the same patient cohort. The concentration of sp-Src varied from 27.46 pg/ml to 919.05 pg/ml, and the cut-off value was 127.38 pg/ml. Accordingly, the patients were divided into high and low-sp-Src groups. Since increased c-Src was negatively correlated with outcomes of advanced NPC patients, subgroup analysis of sp-Src was performed in these subpopulations. Intriguingly, patients with a high level of sp-Src had worse 9-year DFS (*P* = 0.046, Figure 1F) relative to those with low levels of sp-Src in the TNM = IVa-b subgroup, with no significant difference in the baseline clinical characteristics (Supplementary Table S3). Taken together, these data verified the ability of sc-Src and sp-Src levels to predict survival in patients with advanced stages of NPC, though the clinical significance of sp-Src was limited in advanced stage and the statistical significance was marginal.

### Elevated c-Src and p-Src(Y419) in human NPC tissues was correlated with poor outcomes in NPC patients

#### p-Src (Y419) was highly expressed in liver metastasis lesions of NPC patients

**Figure 2:**
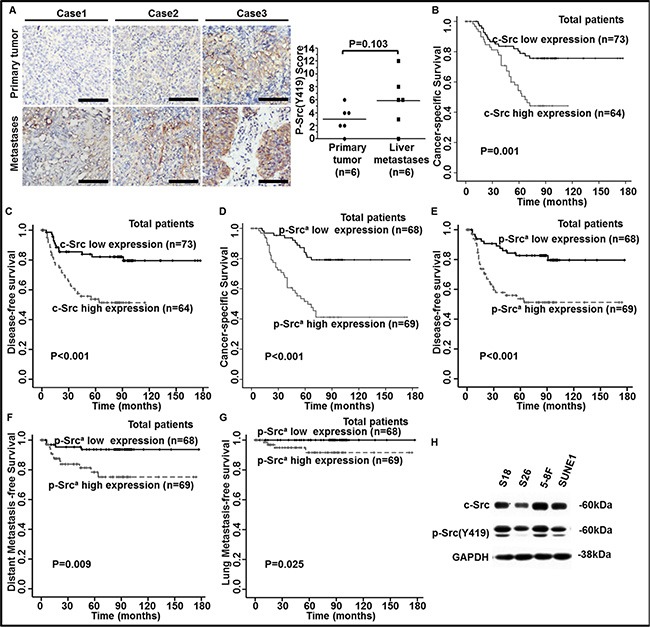
Elevated levels of c-Src and p-Src (Y419) in human NPC tissues were correlated with poor outcomes in NPC patients (**A**) Representative images of IHC staining against p-Src (Y419) in six pairs of human primary NPC tissues and liver metastasis biopsy samples (left panel) and staining scores were shown (right panel). *P* values were calculated using paired *t* test. Scale bars, 50 μm. (**B**–**G**) The levels of c-Src and p-Src (Y419) in 137 NPC patients were accomplished by IHC staining. Kaplan-Meier analysis was used to estimate 15-year cancer-specific survival (CSS), disease-free survival (DFS), distant metastasis-free survival (DMFS), and lung metastasis-free survival (lung-MFS) for NPC patients based on c-Src or p-Src expression in NPC tissues. The 15-year CSS (B) and DFS (C) for the 137 patients, based on c-Src expression, and 15-year CSS (D), DFS (E), DMFS (F) and lung-MFS (G) for the 137 patients, based on p-Src (Y419) expression, were shown. ^a^p-Src, phospho-Src (Y419). *P* values were calculated using unadjusted log-rank tests. (**H**) The expressions of c-Src and p-Src in NPC cell lines (S26, S18, SUNE1 and 5-8F) were determined by western blotting. The expression of glyceraldehyde-3-phosphate dehydrogenase (GAPDH) was used as a loading control.

We speculated that the instability of p-Src, which is easily inactivated in the circulatory system, might explain the limited prognostic value of p-Src in serum at predicting poor outcomes of NPC patients. To evaluate the ability of c-Src and p-Src levels in NPC tissues to predict survival of NPC patients, we quantified p-Src levels in six pairs of primary NPC and liver metastases biopsy samples by immunohistochemistry (IHC) staining. Surprisingly, the liver metastasis samples exhibited higher levels of p-Src compared with their matched primary NPC samples, but without statistical significance (Figure 2A), which may result from small sample size.

#### Up-regulated c-Src and p-Src (Y419) was correlated with poor outcomes of NPC patients

Next, we quantified the levels of c-Src and p-Src via IHC staining in human primary NPC tissues. Patients were divided into c-Src or p-Src high or low groups based on the median values of IHC scores. Patients with high expression of c-Src experienced a lower 15-year CSS and DFS (Figure 2B–2C) compared with patients with low expression of c-Src, with no significant differences in baseline clinical characteristics and treatment strategies between groups (Supplementary Table S4). Moreover, patients with high expression of p-Src experienced a lower 15-year CSS, DFS, DMFS and lung metastasis-free survival (lung-MFS) (Figure 2D–2G) compared with patients with low expression of p-Src.

#### c-Src and p-Src were unfavorable independent factors for NPC patients

After adjusting for the prognostic clinical factors, multivariate analysis verified that the expressions of c-Src in serum or in NPC tissues were independent prognostic factors for 9-year CSS, DFS, DMFS and bone-MFS or 15-year CSS and DFS, respectively. Likewise, p-Src expression in tissues was an independent prognostic factor for 15-year CSS, DFS and DMFS (all *P* < 0.05; Table 1). Altogether, c-Src and p-Src levels in clinical samples could be used to predict the outcomes of NPC patients.

**Table 1:** Multivariate analysis of prognostic factors on survival in ELISA and IHC sets

Patients	Endpoint	Variable	HR	95% CI for HR	*P*^a^
**ELISA set**	CSS	T classification^b^	4.725	2.280–9.791	< 0.001
		N classification^b^	2.399	1.596–3.606	< 0.001
		sc-Src concentration	1.532	1.032–2.274	0.034
	DFS	T classification^b^	2.873	1.687–4.892	< 0.001
		N classification^b^	2.344	1.627–3.376	< 0.001
		sc-Src concentration	1.562	1.092–2.234	0.015
		sp-Src concentration	1.143	0.802–1.629	0.461
	DMFS	T classification^b^	3.334	1.595–6.968	0.001
		N classification^b^	3.870	2.370–6.321	< 0.001
		sc-Src concentration	1.865	1.199–2.900	0.006
		sp-Src concentration	1.152	0.748–1.773	0.520
	bone-MFS	T classificationb	6.694	1.605–27.924	0.009
		N classificationb	4.177	2.050–8.508	< 0.001
		sc-Src concentration	1.988	1.068–3.704	0.030
		sp-Src concentration	1.058	0.581–1.927	0.853
**IHC set**	CSS	c-Src expression	2.217	1.157–4.249	0.016
		p-Src expression	3.720	1.882–7.353	< 0.001
	DFS	c-Src expression	2.465	1.224–4.963	0.011
		p-Src expression	3.295	1.608–6.751	0.001
	DMFS	c-Src expression	1.268	0.468–3.440	0.641
		p-Src expression	3.861	1.227–12.149	0.021

a *P* values were calculated using adjusted Cox proportional hazards model. The following parameters were included in the Cox proportion hazard model by forward (condition): age (continuous variable), gender (male vs. female), T classification (T3–4 vs. T1–2), N classification (N2–3 vs. N0–1), type of histology (WHO III vs II), use of induction chemotherapy (with vs. without), use of chemoradiotherapy (with vs. without), particularly, c-Src concentration/expression and p-Src concentration/expression (high vs. low).

b According to the Union for International Cancer Control/American Joint Committee on Cancer staging system (2002).

Abbreviations: 95% CI, 95% confidence interval; HR, hazard ratio; CSS, cancer-specific survival; DFS, disease-free survival; DMFS, distant metastasis-free survival; bone metastasis-free survival.

To further explore whether c-Src or p-Src correlated with NPC cells' malignancy, we detected the expression of c-Src and p-Src in two pairs of NPC cell lines, including the high-metastasis clones S18 and 5-8F and their corresponding low-metastasis clones S26 and SUNE1. Levels of c-Src and p-Src were consistently higher in S18 than S26, and the p-Src level was higher in 5-8F than SUNE1 even though the difference of c-Src was faint. Hence, we speculated that the high metastasis potential in S18 and 5-8F cells was mainly attributed to highly phosphorylation of c-Src (Figure 2H). Thus, we postulated that p-Src could endow NPC cells with malignant properties, particularly the ability to migrate, resulting in the dissemination of NPC.

### Suppression of p-Src with the SFK inhibitor PP2 inhibited malignant properties of NPC cells

**Figure 3:**
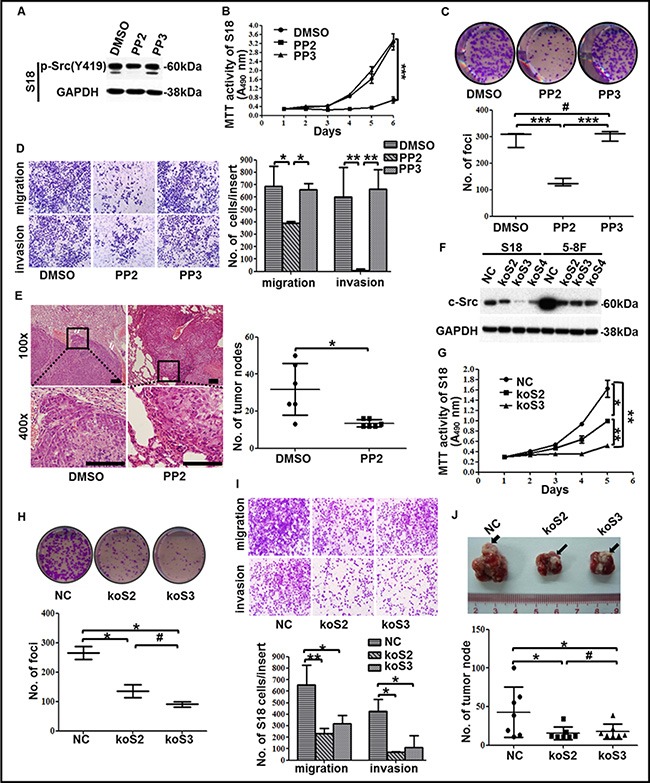
The suppression of c-Src activation or expression in high-metastasis clones inhibited malignancies of NPC (**A**) The levels of p-Src in the high-metastasis clone S18 after incubated with DMSO, PP2 or PP3 were determined by western blotting. GAPDH expression was used as a loading control. (**B**) Cell viability of S18 cells after treatment with DMSO, PP2 or PP3 was shown. (**C**–**D**) Representative images and summaries of colony formation (C) or transwell assays (D) of S18 cells after treatment with DMSO, PP2 or PP3 were shown. (**E**) Representative images of H&E-stained sections derived from lung metastatic nodules of mice injected with S18 cells followed by PP2 or DMSO treatment (left panel). Scale bars, 100 μm. Quantitative analyses of numbers of lung metastatic nodules were shown (*n* = 6, right panel). (**F**) Three sgRNAs targeted c-Src using CRISPR/Cas9 system effectively decreased c-Src expression, as determined by western blotting. A scrambled CRISPR/Cas9 plasmid (NC) was used as the negative control. GAPDH expression was used as a loading control. (**G**) Cell viability of S18 cells after c-Src knockout was shown. (**H**) Representative images and summaries of colony formation of S18 cells after c-Src knockout were shown. (**I**) Representative images and summaries of transwell assays of S18 cells after c-Src knockout were shown. (**J**) Representative images and summaries of lung metastasis nodules derived from nude mice after inoculation with S18-NC or S18-koSrc cells were shown. Metastatic nodules at the surface of the lungs were indicated by arrows (*n* = 6). The indicated values represent means ± SD from at least three independent experiments. One-way ANOVA analyses and Student's *t* tests were used. *P < 0.05; **P < 0.01; ***P < 0.001; ^#^*P* > 0.05 (all *P* values relative to DMSO or PP3 treatment or NC). Different stable cell lines transduced with CRISPR/Cas9 system containing unique sgRNA targeting c-Src were abbreviated to koSrc #2 (koS2), koSrc #3 (koS3) and koSrc #4 (koS4).

To examine the role of p-Src in the regulation of the malignant properties of NPC cells, S18 was incubated with the SFK inhibitor PP2. p-Src was suppressed after incubation with 10 μΜ PP2 for 24 h, but no effect was observed with the negative controls—DMSO or 4-Amino-7-phenylpyrazol[3,4-d]pyrimidine (PP3), a PP2 analog that is inactive on SFKs (Figure 3A). As expected, treatment with PP2 significantly reduced cell viability, colony formation ability, migration and invasion abilities in S18 cells (Figure 3B–3D). Likewise, the suppression of proliferation, colony formation, migration, and invasion was also observed in another high-metastasis clone, 5-8F, when exposed to PP2 (Supplementary Figure S2A–S2D). Although the anti-proliferation effect of PP2 may influence the result of the migration and invasion assays, we noticed that the effect of PP2 on proliferation was not observable until 48 h, whereas the migration and invasion assays were conducted for only 24 h, thus, the influence of proliferation on migration *in vitro* was considered to be limited. Additionally, PP2-induced cell death [32] may also involve the suppression of migration and invasion, therefore, cell death including apoptosis and autophagy were evaluated in NPC cells after PP2 treatment. Notably, PP2 could not induce autophagy in 5-8F until 48 h when compared to DMSO. Moreover, PP2 could only induce cell apoptosis in S18 and 5-8F after 48 h exposure relative to DMSO (Supplementary Figure S2E–S2F), which indicated that the suppression of p-Src activity on migration and invasion was not due to pro-apoptotic effects *in vitro*. Collectively, these results indicated that p-Src may be involved in enhancing survival, proliferation, migration and invasion of NPC cells *in vitro*.

Furthermore, a nude mouse model was used to investigate the effect of PP2 in NPC cells' metastases *in vivo*. Expectedly, PP2 treatment significantly reduced lung metastasis via hematogenous dissemination and erosion into adjacent normal lung tissues caused by NPC cells (Figure 3E). This effect was accompanied by decreasing p-Src (Supplementary Figure S2G), suggesting that PP2-mediated inhibition of c-Src activation could dampen NPC cell metastasis *in vivo*. Taken together, these results verified that p-Src could promote the progression of NPC and suggested that inhibition of p-Src could be a promising strategy for NPC treatment.

### Knockout of c-Src exhibited decreased malignancies of high-metastasis NPC clones *in vitro* and *in vivo*

Because PP2 is an SFK inhibitor, we could not exclude its inhibitory effect on other SFKs, which might have resulted in the aforementioned suppression of NPC malignancies. Therefore, we sought to confirm that it is the inhibition of c-Src activation, not other SFK members, by PP2 that resulted in the depression of malignancies in NPC cells. To this end, we designed three sgRNAs targeting c-Src using CRISPR/Cas9 system (i.e., koSrc #2, koSrc #3, koSrc #4) to knock out c-Src in S18 and 5-8F, which highly express c-Src. A scrambled CRISPR/Cas9 plasmid (NC) was used as a control (Figure 3F). Two of the three c-Src-silenced cell lines (koSrc#2 and koSrc#3) were selected for further research. As expected, the cell viability, colony formation ability, migration and invasion abilities of S18-koSrc cells were significantly decreased relative to S18-NC cells (Figure 3G–3I). Likewise, knocking out c-Src in 5-8F significantly inhibited cell viability, colony formation and migration, but the inhibition of invasion was slight (Supplementary Figure S3A–S3C). This result may indicate the promotion of invasion in 5-8F was only partially mediated by c-Src and is also mediated by other SFKs members, which warrants further investigation.

Additionally, in line with the *in vitro* results, knocking out c-Src significantly reduced lung metastasis *in vivo*, with smaller and fewer metastatic nodules in the lungs of mice after injection of S18-koSrc cells compared with S18-NC cells (Figure 3J and Supplementary Figure S3D). IHC staining against c-Src in the sections derived from the lung metastases verified the absence of c-Src in mice that were injected with S18-koSrc versus those with S18-NC cells (Supplementary Figure S3D). Altogether, these results confirmed that c-Src promoted the proliferation and motility of the high-metastasis clones of NPC *in vitro* and *in vivo*.

### c-Src overexpression significantly enhanced the malignant abilities of the low-metastasis clone S26

**Figure 4:**
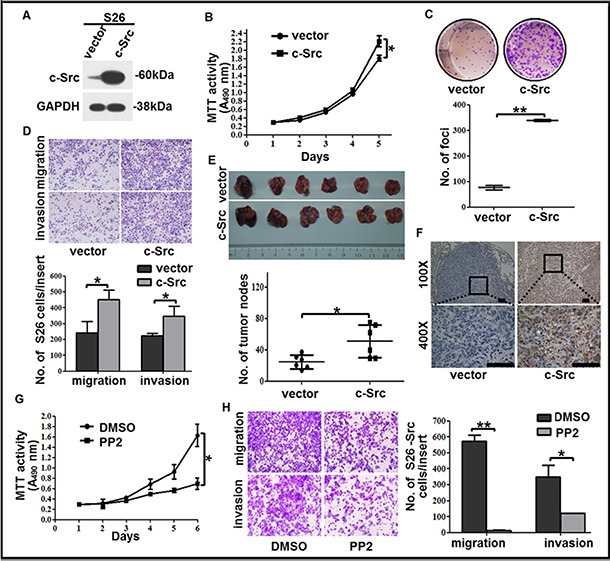
c-Src overexpression increased the proliferation, colony formation, migration and invasion capabilities of the low-metastasis clone S26 (**A**) The level of c-Src was determined by western blotting in S26 cells after c-Src overexpression. GAPDH expression was used as a loading control. (**B**) Cell viability of S26 cells after c-Src overexpression was shown. (**C**) Representative images and summaries of colony formation of S26 cells in monolayer culture after c-Src overexpression were shown. (**D**) Representative images and summaries of migrated and invaded S26 cells after c-Src overexpression were shown. (**E**) Representative images and summaries of lung metastases after injection with S26-vector or S26-Src cells were shown (*n* = 6). (**F**) Continuous sections of mouse lung metastases after injection with S26-vector or S26-Src cells were subjected to IHC staining using an antibody against c-Src. Scale bars, 100 μm. (**G**–**H**) Cell viability (G) and migration or invasion abilities (H) of S26-Src cells after PP2 treatment were shown. The indicated values reflect the means ± SD from at least three independent experiments. Student's *t* test was used for statistical analyses. *P < 0.05; **P < 0.01(all *P* values relative to the S26-vector or DMSO groups).

To determine whether the ectopic expression of c-Src facilitated the low-metastasis clone S26 with increasing malignant properties, we constructed a stable cell line S26-Src by transfecting with ectopic c-Src (Figure 4A). As expected, enhancements of cell viability, colony formation, migration, and invasion were observed in S26-Src compared with the S26-vector negative control (Figure 4B–4D). Likewise, mice injected with S26-Src cells developed more metastatic nodules in the lungs (Figure 4E). IHC staining of c-Src confirmed increased c-Src levels in the metastatic nodules of the lung (Figure 4F). These results verified the role of c-Src in the promotion of NPC cell hematogenous spread and cell survival *in vivo*.

Furthermore, we exposed S26-Src cells to PP2 to explore whether the gain of motility and increased cell viability could be repressed by inhibition of c-Src activation *in vitro*. Expectedly, the proliferation, migration, and invasion abilities of S26-Src were significantly down-regulated when incubated with PP2 relative to DMSO (Figure 4G–4H). These results indicated an increasing effect of motility when overexpressed c-Src in the low metastasis NPC clone, which was mediated by up-regulation of p-Src and could be reversed by PP2.

### c-Src activation promoted NPC metastasis by inducing the EMT process

#### p-Src(Y419) induced the EMT process in NPC cells

**Figure 5:**
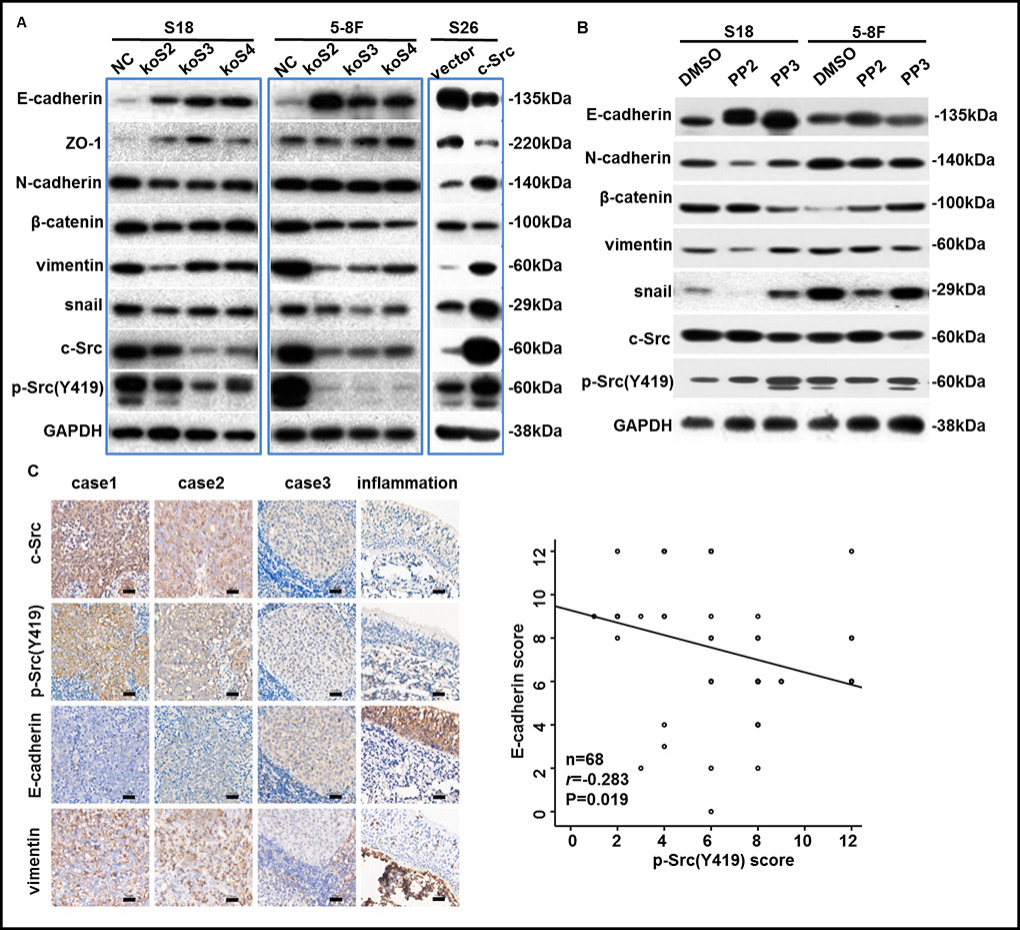
p-Src (Y419) induced the EMT process in NPC cells (**A**–**B**) The levels of c-Src, p-Src and EMT markers in NPC cell lines after knockout, overexpression (A) or inactivation (B) of c-Src were determined by western blotting. GAPDH expression was used as a loading control. (**C**) Representative images of IHC staining with antibodies against c-Src, p-Src (Y419), E-cadherin, and vimentin in human NPC or nasopharyngeal inflammation tissues were shown (left panel). Scale bars, 100 μm. 68 human primary NPC tissue samples that were satisfactorily stained with antibodies against E-cadherin and p-Src (Y419) were scored and plotted (right panel). Spearman correlation analysis was used for statistical analyses. The immunoblots indicated by blue frames were manipulated by enhancing contrast ratio.

To explore whether p-Src promotes NPC cells motility through EMT process, we evaluated the expression of c-Src, p-Src and EMT markers by western blotting in stable cell lines, including S18-koSrc, 5-8F-koSrc and S26-Src, or their parental cell lines after PP2 treatment. Notably, either knocking out c-Src or inactivating p-Src in S18 cells resulted in up-regulation of epithelial-associated markers (E-cadherin and ZO-1) and down-regulation of mesenchymal-associated markers (vimentin and N-cadherin) and EMT-inducing transcription factor snail. Likewise, knocking out c-Src or inactivating p-Src in 5-8F cells increased E-cadherin and reduced snail, but the alterations of ZO-1 and N-cadherin were slight. Vimentin was substantially decreased in c-Src-silenced 5-8F cells, but PP2 treatment did not replicate this decrease (Figure 5A–5B), indicating that the PP2-induced inhibition of motility in 5-8F may only be partially mediated by inhibiting the EMT process. Other mechanisms, including MMP production, cell adhesion, and angiogenesis, could possibly be suppressed when 5-8F cells were exposed to PP2. In contrast, overexpressed c-Src in S26 cells down-regulated E-cadherin and ZO-1 but up-regulated vimentin, N-cadherin and snail compared with the S26-vector. In addition, minimal effect on the expression of β-catenin could be observed in S18 and 5-8F after depletion of c-Src or inactivation of p-Src (Figure 5A–5B). Collectively, these data suggested that c-Src activation promoted NPC cells to metastasis by inducing the EMT process, and the inconsistency in the modulation of different EMT markers was probably due to the specificity of cell lines.

#### p-Src(Y419) negatively correlated with E-cadherin levels in primary NPC tissues

In addition, to further determine the relationship between the levels of c-Src or p-Src and EMT markers in human primary NPC tissues, we evaluated the expression of c-Src, p-Src, E-cadherin and vimentin in 68 human NPC samples as well as an inflammation sample from nasopharynx by IHC. Expectedly, abundant and robust expression of E-cadherin but an absence of vimentin was observed in nasopharyngeal inflammation tissues in which the expression of c-Src or p-Src was almost absent. Spearman correlation analysis confirmed that E-cadherin was significantly negatively correlated with p-Src in human NPC samples, consistent with the results of cell lines (correlation coefficient *r* = −0.283, *P* = 0.019, Figure 5C). Collectively, these results indicated that p-Src-induced NPC progression was likely mediated by inducing the EMT process, which could be restored to some extent by SFK inhibitor PP2.

### c-Src activated the downstream PI3K/Akt pathway to induce migration and invasion in NPC

**Figure 6:**
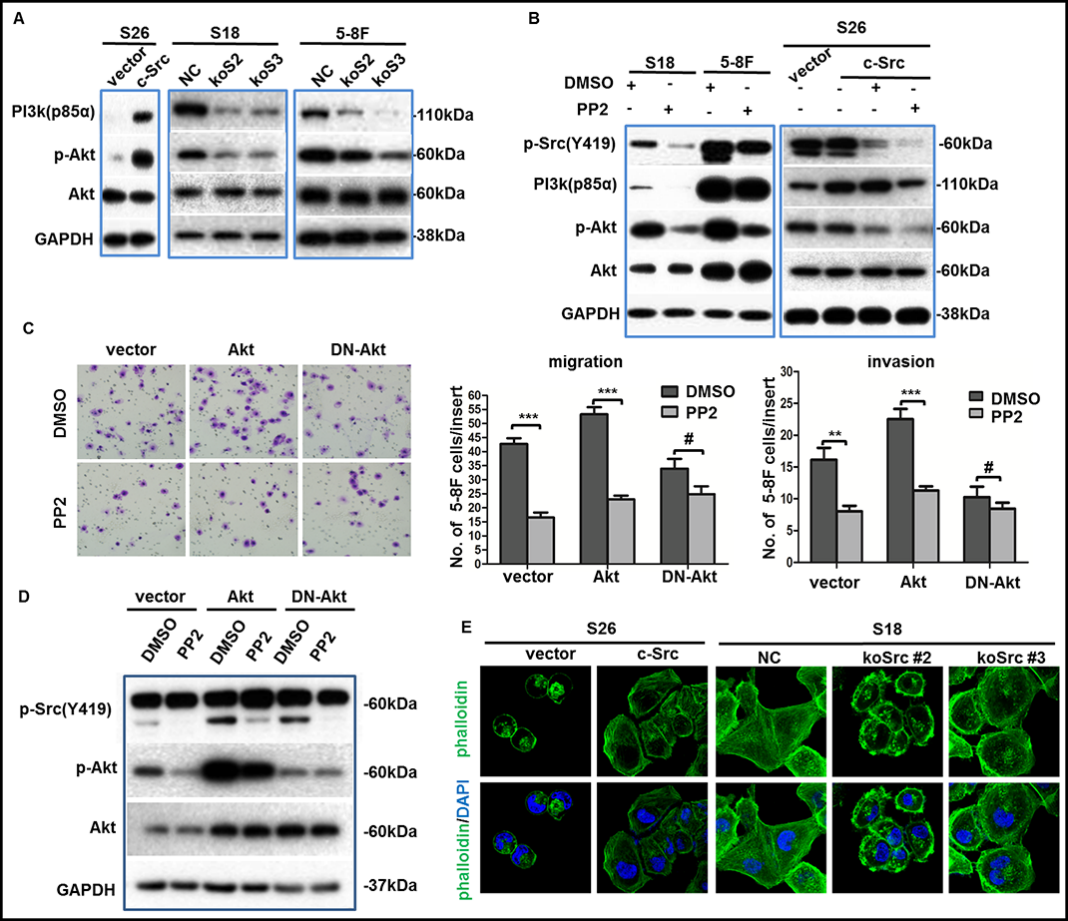
p-Src (Y419) up-regulated the migration and invasion abilities of NPC cells via PI3K/Akt pathway (**A–B**) The phosphorylation levels of PI3K(p85α)/Akt were determined by western blotting in NPC cell lines after overexpression, knockout (A) or inactivation (B) of c-Src. GAPDH expression was used as a loading control. (**C**) Representative images of migrated 5-8F cells transduced with a wild type Akt, a dominant negative Akt (DN-Akt), or a vector control followed by PP2 treatment or not (left panel), summaries of migrated or invaded cells in the lower wells were shown (middle or right panel). The indicated values reflect the means ± SD from at least three independent experiments. Student's *t* test was used for statistical analyses. **P < 0.01; ***P < 0.001; ^#^*P* > 0.05 (all *P* values relative to DMSO). (**D**) Immunoblotting showed the levels of p-Src(Y419), p-Akt, Akt of transduced 5-8F cells after incubation with PP2 or DMSO. GAPDH expression was used as the loading control. (**E**) Representative immunofluorescence images of cytoskeletal in S26 or S18 cells after the overexpression or depletion of c-Src were shown. Nuclei were counterstained with DAPI. The immunoblots indicated by blue frames were manipulated by enhancing contrast ratio.

Due to the central role of p-Src in intracellular signaling transduction, we aimed to determine the predominant downstream signaling pathway of p-Src in NPC cells in order to better understand the pathophysiological process and help determine a useful combination of targeted therapies. Therefore, we evaluated several classical pathways associated with metastasis in S26-Src and S26-vector cells by western blotting, including ERK/MAPK, PI3K/Akt and STAT3. Indeed, the phosphorylation levels of PI3K (p85α) and Akt (Ser473) were stably increased in S26-Src relative to the S26-vector but down-regulated in S18-koSrc and 5-8F-koSrc cells compared with S18-NC and 5-8F-NC cells (Figure 6A). However, alterations of the other two pathways could not reach a consistent conclusion with overexpressed and silenced c-Src in NPC metastasis. To further verify the role of p-Src in stimulating the PI3K/Akt signaling pathway in NPC cells, the phosphorylation levels of PI3K/Akt were evaluated in S18, 5-8F and S26-Src/S26-vector cells after incubation with PP2 for 24 h. Consistent with the aforementioned results, the levels of PI3K (p85α) and p-Akt were dramatically repressed following PP2 treatment (Figure 6B). Particularly, growing evidence has revealed a key role of PI3K/Akt–NF-κB–Snail in regulating the EMT process in many other malignancies [33]. To further confirm the involvement of PI3K/Akt pathway in inducing metastasis of NPC, we first transfected 5-8F cells with a wild type Akt, a dominant negative Akt (DN-Akt), or a vector control, which followed by PP2 or DMSO treatment. Then migration and invasion abilities were determined by transwell assays *in vitro*. As expected, DN-Akt could inhibit the migration and invasion abilities in 5-8F cells. DN-Akt also abolished the PP2-induced suppression of migration and invasion abilities (Figure 6C). Western blotting confirmed that the phosphorylated level of Akt was decreased, but no PP2-induced down-regulation of p-Akt was obtained in DN-Akt transduced 5-8F cells, when relative to vector or wild type Akt transduced cells (Figure 6D). Altogether, we could infer that p-Src promote NPC cell metastasis via activating the PI3K/Akt pathway, which sequentially inducing the EMT process.

Additionally, rearrangement of the cytoskeleton in malignant cancer cells is involved in the metastatic process, and c-Src has been shown to regulate cytoskeleton proteins [34–36]. However, the effect of c-Src in the cytoskeletal remodeling of NPC cells has seldom been studied. Here, we performed immunofluorescence analyses in S26-Src and S18-koSrc cells to observe the cytoskeletons of these cells. Notably, the cytoskeleton of S26-Src cells became angular and well organized relative to the S26-vector cells. Conversely, the cytoskeleton of S18-koSrc cells disintegrated and cells turned out to be round compared with S18-NC cells (Figure 6E). These observations strongly indicated that c-Src was involved in the arrangement of the cytoskeleton in NPC cells.

## DISCUSSION

The correlation between c-Src expression or activation in tumor tissues and clinical prognosis has been extensively studied in many malignancies. Previously, Gallick *et al*. verified that increased c-Src activities and protein levels were correlated with progression of primary colon carcinomas to liver metastases in two independent studies [37, 38], and they also suggested that c-Src activity was an independent indicator of poor outcomes in all stages of human colon carcinoma [39]. Src kinase had the highest expression level of the SFKs and correlated with poor DFS in breast cancer [40], as well as poor overall survival and recurrence survival rates in cervical cancer [41]. Herein are the first results to reveal the clinical significance of levels of sc-Src in serum and p-Src in primary NPC tissues for DFS in local advanced NPC patients. The prognostic value of sc-Src and sp-Src protein in serum makes evaluation simpler and less invasive. However, since the time span between serum sample collection and detection is long enough to suffer from degradation or inactivation of p-Src, the prognostic value of p-Src may need to be verified in further trials. Furthermore, the correlation of autoantibodies against p-Src and NPC outcomes should be tested as an alternative because autoantibodies are more stable than p-Src per se.

Additionally, recent studies have demonstrated that p-Src correlated with distant metastases and aggressiveness in malignant pleural mesothelioma [42], prostate cancer [43, 44], and esophageal cancer [45]. Consistently, both levels of sc-Src and p-Src in primary NPC tissues were unfavorable prognosis factors for DMFS of NPC patients. Moreover, the correlations between levels of p-Src in tissues and lung-MFS or sc-Src and bone-MFS indicated that c-Src activation may induce the tropism of NPC cells to lung and bone. Particularly, increased c-Src expression or activation in NPC cells could promote lung metastasis in nude mice models via tail vein injection. Consistently, c-Src activity associated with tumor colonization of bone and lung in an animal model in which MDA-231 human breast cancer cells were inoculated into the left cardiac ventricle [46]. Altogether, although sp-Src levels provided limited prognostic value, highly expressed and activated c-Src in serum and NPC tissues could still be a prognostic biomarker for patients with local advanced NPC, especially in predicting distant metastasis.

It's reported that c-Src was a well characterized pro-oncogene involved in key cellular events and tumor progression [47]. And its clinical significance also suggested a biological role of c-Src in the malignant properties of NPC. Expectedly, c-Src activation promoted the abilities of cell proliferation, motility, invasion and anti-apoptotic in NPC cells. Indeed, there has been increasing interest in the pro-metastatic potential of p-Src in cancers during the past decade. For instance, the recruitment of c-Src to EGFR and EGFR-induced phosphorylation of c-Src could be inhibited by NDRG1, the potent metastasis suppressor N-myc downstream regulated gene-1. And this suppression could result in inhibition of cancer metastasis in colon and pancreatic cancer cell lines [48]. Similarly, Zhang *et al*. reported that dermatopontin (DPT) could inhibit hepatocellular cell motility by reducing focal adhesion kinase and c-Src phosphorylation [49]. Likewise, depletion or inactivation of c-Src could reduce migration *in vitro* and metastasis *in vivo* of NPC cells and these malignancies could be restored by overexpression of c-Src. Additionally, the clinical prognostic value of p-Src for DMFS of NPC patients and evidence of higher levels of p-Src (Y419) in liver metastases relative to paired primary NPC tissues were also revealed in this study. Altogether, these results suggested that c-Src activation could promote the metastasis of NPC and be used as a promising therapeutic target in NPC patients. Notably, a recent publication has verified that dasatinib, an orally available tyrosine kinase inhibitor (TKI), could inhibit the migration of NPC cell line CNE2 *in vitro* by repressing the phosphorylation of c-Src and its downstream molecules AKT, MEK, ERK in a dose-dependent manner [50], which further confirmed the therapeutic value of p-Src in NPC patients.

Notably, EMT has been recognized to be vital in promoting cancer metastasis [51], and c-Src has been demonstrated to play a crucial role in the induction of E-cadherin repressors and the vimentin promoter, which allows tumor cells to migrate/invade by activating downstream signaling pathways [26, 52, 53]. Here, we showed that c-Src activation promoted the EMT process by activating the downstream PI3K/Akt pathway in NPC cells. Although there were some discrepancies in the p-Src-dependent alteration of the EMT markers in different cell lines, the trends of the EMT markers remained consistent. For example, down-regulated p-Src increased the epithelial-associated markers and decreased the mesenchyme-associated markers in both S18 and 5-8F cell lines, leading to inhibition of cancer metastasis. These results supported the role of p-Src in inducing the EMT process in NPC. Furthermore, the results demonstrate the complexity of the EMT process and its cell-type specificity, of which detailed mechanisms should be further determined. Of note, PP2 dramatically suppressed the p-Src-dependent malignant abilities of NPC cells *in vitro* and *in vivo* via a retarded EMT process, which was possibly mediated by blocking the PI3K/Akt signaling pathway. And this mechanism of PP2 in reducing cancer metastasis was previously reported in colon and breast cancer cells [54]. Accordingly, inhibition of c-Src activation in NPC could suppress metastatic potential and act as a novel targeted therapeutic strategy for patients with NPC, particularly for patients in advanced diseases.

In addition, cytoskeleton remodeling could be observed in cancer cells and contributes to cell migration and invasion. Up-regulation of the cytoskeletal protein cortactin/EMS1, a relatively specific substrate of c-Src [34–36], has been reported to be involved in metastases [55] and correlated with poor outcomes of patients with breast cancer [56, 57]. Moreover, c-Src has been revealed to affect the downstream effector RhoA, which could induce cytoskeleton remodeling [58]. Notably, the cytoskeleton of NPC cells was remodeled concomitantly with c-Src alterations in our study, indicating that c-Src-induced cytoskeletal remodeling similarly endowed NPC cells with high potential of metastasis.

In summary, our study suggested that c-Src activation might be a novel biomarker and a promising therapeutic target for NPC patients at high risk for metastasis. Therefore, identifying patients at high-risk using this biomarker, and treating those found to be with this corresponding targeted therapy could maximize benefits. However, further prospective clinical trials of c-Src tyrosine kinase inhibitors in selected population of NPC patients will be highly informative.

## MATERIALS AND METHODS

Detailed procedures are provided in the Supplementary Experimental Procedures.

### Clinical characteristics of patients and tissue microarrays

All human serum samples were obtained from the cancer repository and human tissue samples obtained from the Department of Pathology of the Sun Yat-sen University Cancer Center (SYSUCC) with prior patient consent and the approval of the Institutional Clinical Ethics Review Board at SYSUCC. The serum samples used for ELISA were collected from 290 patients between June 1st, 2003 and August 30th, 2005, with a median age of 44 years (range, 18 to 65 years), and the tissue microarrays (TMAs) contained 137 qualified primary NPC samples between December 1st, 1998 and December 31st, 1999, with a median age of 47 years (range, 18 to 71 years). Detailed clinical characteristics of patients of both cohorts were listed in Supplementary Tables S1 and S4. The median follow-up duration of patients in both cohorts was 80 months (range, 8–105 months) and 72 months (range, 1–177 months), respectively. 106 and 49 patients died during follow-up, including 101 and 48 patients died from cancer progression in ELISA and IHC cohorts, respectively. TMAs were constructed as described previously [59]. The six pairs of primary NPC and liver metastasis samples were from six NPC patients with liver metastases at diagnosis. All patients were pathologically diagnosed patients at SYSUCC.

### Immunohistochemical staining (IHC)

The paraffin-embedded blocks were sectioned for IHC staining as performed previously [60]. Briefly, paraffin-embedded tissue specimens were deparaffinized and rehydrated. Antigenic retrieval was processed with sodium citrate (p-Src[Y419], E-cadherin and vimentin) or 0.5 M EDTA (c-Src) using a microwave at 700 W for 10 minutes. The sections were then incubated in H2O2 (3%) for 10 min, blocked in goat serum at room temperature for 30 min and incubated with anti-c-Src (1:400), p-Src(1:200), anti-E-cadherin (1:500) or anti-vimentin (1:500) antibodies overnight at 4°C. An EnVision kit (DAKO, Carpinteria, CA, USA) was used to detect the primary antibodies followed by 3,3-diaminobenzidine substrate visualization and counterstaining with hematoxylin. The intensity of IHC staining in the tumor cells was scored independently by two pathologists using the semiquantitative immunoreactive score (IRS) scale (for staining intensity, no staining = 0; weak staining = 1; moderate staining = 2; strong staining = 3; for the percentage of stained cells, 0% = 0; 1%–10% = 1; 11%–50% = 2; 51%–80% = 3; 81%–100% = 4) [61]. The average values from the two pathologists were used for further analyses. The median and range of the IRS for each antibody in this study were listed in Supplementary Table S5.

### Cell culture and PP2 treatment

The subclones of human NPC cell line CNE-2 (S18 and S26), SUNE-1 and its subclone 5-8F, and 293T cells (all cell lines cultured for less than 50 passages, presented by Qian CN) were maintained in Dulbecco's modified Eagle's medium supplemented with 10% FBS. PP2 (Calbiochem, La Jolla, CA, USA) was stored as a stock solution in 100% DMSO (Sigma-Aldrich, St. Louis, MO, USA) at a concentration of 1 mg/ml. For the PP2 inhibition section, the cells were incubated with PP2 (10^−5^ M), PP3 (10^−5^ M) or the same volume of DMSO (0.1%) as a blank control from day 1 in MTT and colony formation assays or for 24 h before transwell assays; the medium was replaced every three days when necessary.

### MTT and colony formation assays

For the MTT assay, S18, 5-8F and S26 cells were seeded at a density of 1,200 cells per well in 96-well plates at day 0. The absorbance values were measured at 490 nm using an ELX800 spectrophotometric plate reader (Bio-Tek, Winooski, VT, USA) from day 1 to day 5 or 6 (PP2 treatment section). For the colony-formation assay, the cells were plated at a density of 500 cells per well in six-well plates and cultured for 14 days. The colonies were fixed in methanol, stained with 0.5% gentian violet and counted.

### Analysis of apoptosis by flow cytometry

Apoptotic cells after intended treatment were collected, washed with PBS, stained with FITC-conjugated Annexin V and propidium iodide (PI) according to the manufacturer's instructions using a commercially apoptosis detection kit (Biosea, Beijing, China). Then cells were analyzed by FACS Calibur flow cytometer (Beckman Coulter Corp., CA, USA).

### *In vitro* migration and invasion assays

Invasion and migration assays were performed in Transwell chambers (Corning, Tewksbury, MA, USA) coated with or without Matrigel (BD Biosciences, San Diego, CA, USA) on the surface of upper chamber. Briefly, S18 and 5-8F cells or stably transfected cells were harvested and resuspended in serum-free medium. Cells (for S18 and 5–8F, 4 × 10^4^ or 2 × 10^4^ and for S26, 8 × 10^5^ or 4 × 10^4^, respectively) were plated into the upper chamber for the invasion or migration assays. Medium supplemented with 10% FBS was placed in the lower chamber. After 24 h incubation, the cells that had invaded or migrated through the membrane to the lower surface were fixed, stained and counted using an inverted microscope.

### *In vivo* metastasis study

BALB/c nude mice, at age 4–5 weeks, were provided by the Medical Experimental Animal Center of Guangdong Province (Guangzhou, China) and fed in a specific pathogen-free environment. The mice were randomly grouped into each experimental group (*n* = 6). Briefly, 10^6^ viable cancer cells resuspended in 150 μl normal saline were injected into the mice via the tail vein. For PP2 treatment, PP2 (5 mg/kg/day) in a 1% DMSO vehicle at a volume of 0.10 ml solution/10 g body weight was administered daily for three weeks from the day after cell inoculation via an intraperitoneal route. The control group received the same volume as the 1% DMSO vehicle. The mice were sacrificed at the end of the 6th week after inoculation, necropsies were performed immediately, and metastatic nodules were counted. All animal research protocols were approved by the Institutional Animal Care and Use Ethics Committee of SYSUCC and were in accordance with national guidelines for the care and maintenance of laboratory animals.

### Statistical analyses

All analyses were performed with SPSS 19.0 software. The receiver operating characteristic (ROC) curve analysis was performed to establish the cut-off values for the concentrations of c-Src and p-Src related to the events of recurrence or distant metastasis. The median IHC score value was used as the cut-off value to divide the patients into high- and low-c-Src or p-Src(Y419) expression groups. Comparisons between groups were analyzed by Student's *t* tests (paired or independent) or one-way ANOVAs and chi-squared or Fisher exact tests, if indicated. Spearman rank correlation analyses was used for correlation analyses. Survival duration was calculated from the first day of diagnosis of NPC. Actuarial rates of survival were calculated using the Kaplan–Meier method, and differences were compared using log-rank tests. Multivariate analyses using an adjusted Cox proportional hazards model were conducted to identify significant independent variables with a forward (condition) method. A 2-tailed *P* value less than 0.05 was considered significant.

### Accession numbers

The Gene Expression Omnibus database accession number for the protein microarray data reported in this paper is GSE68764.

## SUPPLEMENTARY MATERIALS FIGURES AND TABLES


