# Supplementary material for: c-Src activation promotes nasopharyngeal carcinoma metastasis by inducing the epithelial-mesenchymal transition via PI3K/Akt signaling pathway: a new and promising target for NPC
Source: Oncotarget. 2016 Apr 7;7(19):28340–55. doi: 10.18632/oncotarget.8634 (PMC5053730; doi:10.18632/oncotarget.8634)
Supplement: Supplementary file 1 [file oncotarget-07-28340-s001.pdf]

c-Src activation promotes nasopharyngeal carcinoma metastasis by inducing the epithelial-mesenchymal transition via PI3K/Akt signaling pathway: a new and promising target for NPC

Supplementary Materials

Antibodies

The following antibodies were used: c-Src, p-Src(Y419), N-cadherin, ZO-1, snail, p-Akt, GAPDH (Cell Signaling Technology, Beverly, MA, USA, cat#2109, 2101,14215, 8193, 3879, 4060, 2118, respectively); E-cadherin, vimentin, β-catenin, Akt (BD Biosciences, San Diego, CA, USA, cat#610182, 550513, 610153, 610836, respectively); PI3K(p85α), LC3 (Proteintech, Chicago, IL, USA, cat#60225-1, 14600-1-AP, respectively); and phalloidin (Sigma-Aldrich, St Louis, MO, USA, cat#P5282).

ELISA analyses of levels of sc-Src and sp-Src(Y419) in the serum of NPC patients

The concentrations of sc-Src and sp-Src(Y419) in serum were measured using commercially available human sc-Src and sp-Src(Y419) quantitative ELISA kits (R&D Systems, Minneapolis, MN, USA and Cell Signaling Technology, MA, USA) according to the manufacturers' protocols. The values were measured with an enzyme-linked spectrophotometer at a wavelength of 450 nm after the reaction. The concentrations of sc-Src and sp-Src(Y419) were calculated separately from standard curves. The standard curves of the kits ranged from 0 pg/ml to 2000 pg/ml. All analyses were performed in duplicate, and the mean values were used for statistical analysis.

Plasmids and stably transfected cell lines

The sgRNAs targeting c-Src were designed as reported [1] using the tools described at http://tools.genomeengineering.org. Three guided sequences with minimal off-target gene targeting and high on-target scores were selected. The lentiviral containing sgRNA targeting c-Src and CRISPR/Cas9 system was constructed using the lentiviral CRISPR toolbox (Addgene, Cambridge, MA, USA) according to the manufacturer's protocol [1]. Empty vector were used as controls. The oligos (Invitrogen) used to generate the guided sequences were as follows: c-Src-g-F2 (5′-CACCGCCCTCTATGACTATGAGTCT-3′) and c-Src-g-R2 (5′-AAACAGACTCATAGTCATAGAGGG-3′), c-Src-g-F3 (5′-CACCGTAACCGCTCTGACTCCCGTC-3′) and c-Src-g-R3 (5′-AAACGACGGGAGTCAGAGCGGTTA-3′), c-Src-g-F4 (5′-CACCGAGCGCCGTGCACGTTCTCGG-3′) and c-Src-g -R4 (5′-AAACCCGAGAACGTGCACGGCGCT-3′).

c-Src sequence was cloned into the lentiviral pBabe-puro expression vector (Invitrogen). pBabe-puro was used as a negative control. Briefly, 293T cells were transfected with intended plasmids using Lipofectamine 2000 reagent (Invitrogen) according to the manufacturer's instructions on day1. Virus was collected and infected target cells on day 3 and day 4. Stably transfected cells were selected for one week using puromycin (final concentration, 0.5 μg/mL).

The dominant negative Akt (DN-Akt) was cloned from wild-type Akt with mutation at K179M/T308A/S473A and ligated into pcDNA3.1 using Infusion kits (Clontech). The 5-8F cells were transiently transfected with DN-Akt, wild type Akt or pcDNA3.1. The oligonucleotides used to generate point mutations were as followed: 5′-aggccgctactacgccatgatgatcctcaagaagg-3′/5′-gcgtagtagcgggcctgtggc cttctccttcaccag-3′ (K179M), 5′-cggtg ccaccatgaaggccttttgc ggcacacctg-3′/5′-ttcatggtggcaccgtccttgatcccctccttgca (T308A), 5′-ccacttcccc cagttcGcct actcggccag cggca-3′/5′-aactgggggaagtggggcctgcgctcgctgtccac-3′(S473A).

Western blot analysis

Cells were lysed on ice in RIPA buffer containing protease and phosphatase inhibitors (Fdbio Science, Hangzhou, China), and the relative protein concentrations were determined using Coomassie Brilliant Blue. Total proteins were separated on 10% SDS-PAGE gels and transferred to polyvinylidene fluoride membranes (Merck Millipore, Billerica, MA, USA), and the membranes were incubated with the following antibodies overnight at 4°C: anti-c-Src (1:1,000); anti-p-Src (Y419; 1:1,000); anti-E-cadherin (1:1,000); anti-vimentin (1:1,000); anti-N-cadherin (1:1,000); anti-β-catenin (1:5,000); anti-ZO-1 (1:1,000); anti-snail (1:1,000); anti-p-Akt (1:1,000); anti-Akt (1:1,000); anti-PI3K (p85α; 1:1,000); anti-LC3 (1:1,000); anti-GAPDH (1:3,000). Afterwards,

the membranes were incubated with anti-rabbit or anti-mouse IgG secondary antibodies (1:20,000; Epitomics, Burlingame, CA, USA) for 1 h at room temperature after removing the unbound primary antibodies. The bands were detected by enhanced chemiluminescence.

Immunofluorescent labeling and confocal microscopy

The cytoskeleton of cells was viewed by direct immunofluorescence. Briefly, cells grown on slides were fixed with 4% paraformaldehyde at room temperature for 30 min and permeabilized in 0.5% Triton X-100 in PBS for 20 min. The slides were incubated with phalloidin antibodies conjugated with FITC (1:1000, green). The nuclei were stained with DAPI (Sigma-Aldrich, 1:1000, blue). An Olympus IX71 laser scanning confocal microscope was used for microscopic analyses, and images were scanned according to the manufacturer's instruction.

REFERENCES

1. Ran FA, Hsu PD, Wright J, Agarwala V, Scott DA, Zhang F. Genome engineering using the CRISPR-Cas9 system. 2013; 8:2281–2308.

Supplementary Table S1: Clinical characteristics of NPC patients that sc-Src level was detected in serum by ELISA

Characteristics	All	sc-Src concentration		P^a
		Low	High	
	No (%)	No (%)	No (%)	
**Age, years**	290	163	127	0.387
≤ 50	224 (77.2)	121 (74.2)	103 (81.1)	
> 50	66 (22.8)	42 (25.8)	24 (18.9)	
**Sex**				0.387
Male	204 (70.3)	118 (72.4)	86 (67.7)	
Female	86 (29.7)	45 (27.6)	41 (32.3)	
**Histology, WHO type**[b]				0.783
II	36 (12.4)	21 (12.9)	15 (11.8)	
III	254 (87.6)	142 (87.1)	112 (88.2)	
**T stage**[c]				0.005
1/2	79 (27.2)	55 (33.7)	24 (18.9)	
3/4	211 (72.8)	108 (66.3)	103 (81.1)	
**N stage**[c]				0.754
0/1	156 (53.8)	89 (54.6)	67 (52.8)	
2/3	134 (46.2)	74 (45.4)	60 (47.2)	
**Clinical stage**[c]				0.065
I/II[d]	50 (17.2)	34 (20.9)	16 (12.6)	
III/IVa-b[d]	240 (82.8)	129 (79.1)	111 (87.4)	
**Treatment**				0.053
RT alone	53 (18.3)	35 (21.5)	18 (14.2)	
ICT + RT	90 (31.0)	57 (35.0)	33 (16.0)	
CRT	56 (19.3)	27 (16.6)	29 (22.8)	
ICT + CRT	91 (31.4)	44 (27.0)	47 (37.0)	

[a]*P* values were calculated using the chi-square test or Fisher exact test if indicated.
[b]II, differentiated nonkeractinizing carcinoma; III, undifferentiated nonkeractinizing carcinoma.
[c]According to the Union for International Cancer Control/American Joint Committee on Cancer staging system (2002).
[d]I, T1N0M0; II, T2N0-1M0, T1N1M0; III, T3N0-2M0, T1-2N2M0; IVa-b, T4N0-3M0, T1-3N3M0.
Abbreviations: RT, radiotherapy; CRT, chemoradiotherapy; ICT, Induction chemotherapy.

Supplementary Table S2: Clinical characteristics of NPC patients with T stage = 3/4[a] disease that sc-Src level was detected in serum by ELISA

	All	sc-Src concentration		
		Low	High	P^b
Characteristics	No (%)	No (%)	No (%)	
**Age, years**	211	108	103	0.115
≤ 50	160 (75.8)	77 (71.3)	83 (80.6)	
> 50	51 (24.2)	31 (28.7)	20 (19.4)	
**Sex**				0.330
Male	142 (67.3)	76 (70.4)	66 (64.1)	
Female	69 (32.7)	32 (29.6)	37 (35.9)	
**Histology, WHO type[c]**				0.498
II	28(13.3)	16 (14.8)	12 (11.7)	
III	183 (86.7)	92 (85.2)	91 (88.3)	
**N stage[d]**				0.838
0/1	106 (50.2)	55 (50.9)	51 (49.5)	
2/3	105 (49.8)	53 (49.1)	52 (50.5)	
**Treatment**				0.144
RT alone	4 (1.9)	2 (1.9)	2 (1.9)	
ICT + RT	76 (36.0)	47 (43.5)	29 (28.2)	
CRT	47 (22.3)	21 (19.4)	26 (25.2)	
ICT + CRT	84 (39.8)	38 (35.2)	46 (44.7)	

[a]T3-4N0-3M0.
[b]*P* values were calculated using the chi-square test or Fisher exact test if indicated.
[c]II, differentiated nonkeractinizing carcinoma; III, undifferentiated nonkeractinizing carcinoma.
[d]According to the Union for International Cancer Control/American Joint Committee on Cancer staging system (2002).
Abbreviations: RT, radiotheropy; CRT, chemoradiotherapy; ICT, Induction chemotherapy.

Supplementary Table S3: Clinical characteristics of NPC patients with clinical stage = IVa-b[a] disease that sp-Src level was detected in serum by ELISA

	All	sp-Src concentration		P^{b}
		Low	High	
Characteristics	No (%)	No (%)	No (%)	
**Age, years**	109	60	49	0.311
≤ 50	87 (79.8)	50 (83.3)	37 (75.5)	
> 50	22 (20.2)	10 (16.7)	12 (24.5)	
**Sex**				0.185
Male	76 (69.7)	45 (75.0)	31 (63.3)	
Female	33 (30.3)	15 (25.0)	18 (36.7)	
**Histology, WHO type[c]**				0.075
II	17 (15.6)	6 (10.0)	11 (22.4)	
III	92 (84.4)	54 (90.0)	38 (77.6)	
**T stage[d]**				0.405
1/2	6 (5.5)	2 (3.3)	4 (8.2)	
3/4	103 (94.5)	58 (96.7)	45 (91.8)	
**N stage[d]**				0.360
0/1	52 (47.7)	31 (51.7)	21 (42.9)	
2/3	57 (52.3)	29 (48.3)	28 (57.1)	
**Treatment**				0.632
RT alone	0 (0.0)	0 (0.0)	0 (0.0)	
ICT + RT	42 (38.5)	24 (40.0)	18 (36.7)	
CRT	14 (12.8)	9 (15.0)	5 (10.2)	
ICT + CRT	53 (48.6)	27 (45.0)	26 (53.1)	

[a]IVa–b, T4N0-3M0, T1-3N3M0.
[b]*P* values were calculated using the chi-square test or Fisher exact test if indicated.
[c]II, differentiated nonkeractinizing carcinoma; III, undifferentiated nonkeractinizing carcinoma.
[d]According to the Union for International Cancer Control/American Joint Committee on Cancer staging system (2002).
Abbreviations: RT, radiotheropy; CRT, chemoradiotherapy; ICT, Induction chemotherapy.

Supplementary Table S4: Clinical characteristics of NPC patients that c-Src and p-Src expressions were quantified in primary NPC tissues by IHC staining

	All	c-Src expression			p-Src expression		
		Low	High	P^a	Low	High	P^a
Characteristics	No (%)	No (%)	No (%)		No (%)	No (%)	
**Age, years**	137	73	64	0.770	68	69	0.053
≤ 50	86 (62.8)	45 (61.6)	41 (64.1)		37 (54.4)	49 (71.0)	
> 50	39 (37.2)	28 (38.4)	23 (35.9)		31 (45.6)	20 (29.0)	
**Sex**				0.517			0.800
Male	108 (78.8)	56 (76.7)	52 (81.3)		53 (77.9)	55 (79.7)	
Female	29 (21.2)	17 (23.3)	12 (18.8)		15 (22.1)	14 (20.3)	
**Histology**[b]				0.750			0.532
II	10 (7.3)	6 (8.2)	4 (6.2)		6 (8.8)	4 (5.8)	
III	127 (92.7)	67 (91.8)	60 (93.8)		62 (91.2)	65 (94.2)	
**T stage**[c]				0.121			0.141
1/2	76 (55.5)	45 (61.6)	31 (48.4)		42 (61.8)	34 (49.3)	
3/4	61 (44.5)	28 (38.4)	33 (51.6)		26 (38.2)	75 (50.7)	
**N stage**[c]				0.109			0.372
0/1	98 (71.5)	48 (65.8)	50 (78.1)		51 (75.0)	47 (68.1)	
2/3	39 (28.5)	25 (34.2)	14 (21.9)		17 (25.0)	22 (31.9)	
**Clinical stage**[c]				0.770			0.144
I/II[d]	56 (40.9)	29 (39.7)	27 (42.2)		32 (47.1)	24 (34.8)	
III/IVa-b[d]	81 (59.1)	44 (60.3)	37 (57.8)		36 (52.9)	45 (65.2)	
**Treatment**[e]				0.522			0.445
RT alone	98 (71.5)	50 (68.5)	48 (75.0)		46 (67.7)	52 (75.38)	
CRT	10 (7.3)	6 (8.2)	4 (6.3)		6 (8.8)	4 (5.8)	
ICT + CRT	5 (15.3)	14 (19.2)	7 (10.9)		11 (16.2)	10 (14.5)	
ICT + RT	21 (3.7)	2 (2.7)	3 (4.7)		4 (5.9)	1 (1.5)	

[a]*P* values were calculated using the chi-square test or Fisher exact test if indicated.
[b]II, differentiated nonkeractinizing carcinoma, III, undifferentiated nonkeractinizing carcinoma.
[c]According to the Union for International Cancer Control/American Joint Committee on Cancer staging system (2002).
[d]I, T1N0M0; II,T2N0-1M0, T1N1M0; III, T3N0-2M0, T1-2N2M0; IVa-b, T4N0-3M0, T1-3N3M0.
[e]The treatment information of three patients was unavailable.
Abbreviations: RT, radiotheropy; ICT, Induction chemotherapy; CRT, chemoradiotherapy.

Supplementary Table S5: Median and range of IRS for each antibody

		c-Src		p-Src(Y419)		e-cadherin	vimentin
group		low	high	low	high	-	-
median		4	9	4	8	-	-
range		0–6	8–12	1–6	8–12	-	-
total	median	6		8		6	0
	range	0–12		1–12		0–12	0–8

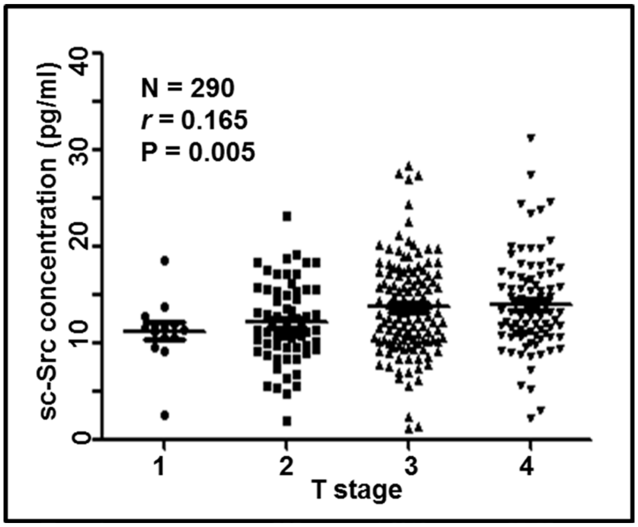

Supplementary Figure S1: The scatter plot of sc-Src concentration in patients with different T stages was shown. Spearman correlation analysis was used for statistical analyses.

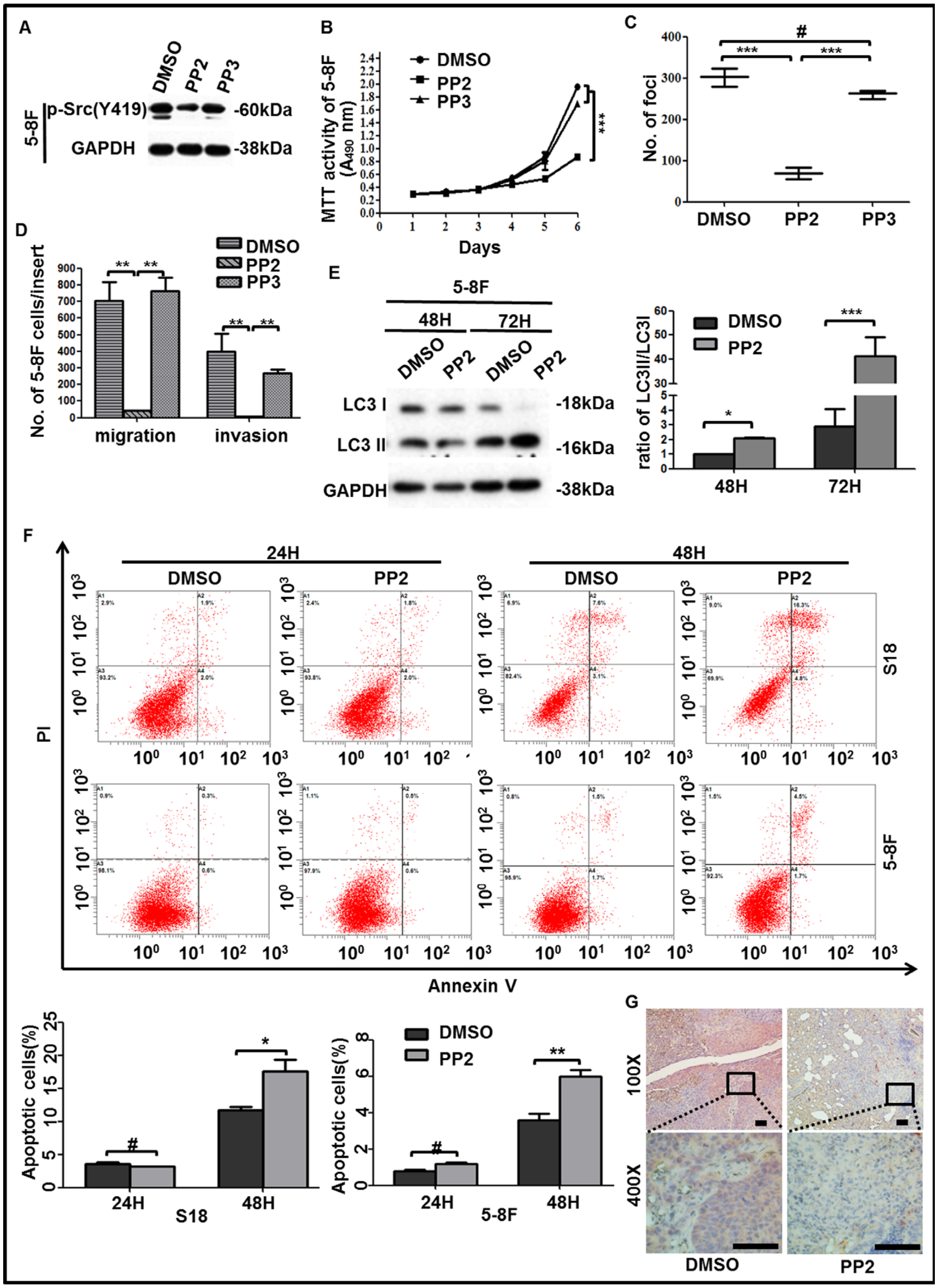

Supplementary Figure S2: The suppression of c-Src activation or expression in high-metastasis clones inhibited NPC cells malignant capabilities. (**A**), c-Src activation decreased in the high-metastasis clone 5-8F after PP2 treatment, as determined by western blotting. DMSO and PP3 were used as the blank and negative controls, respectively; GAPDH was used as a loading control. PP2 decreased the viability (**B**), colony formation ability (**C**), migration and invasion abilities (**D**) of 5-8F cells. (**E**), 5-8F cells were treated with PP2 or DMSO for 48 h or 72 h, expression of LC3 type I and II were detected by WB (left panel), ratios of type II to I were calculated (right panel). GAPDH was used as the loading control. (**F**), S18 and 5-8F cells were treated with PP2 or DMSO for 24 h or 48 h, apoptotic cells were measured by FACS analysis. One-way ANOVA analyses and Student's *t* test were used for statistical analyses. **P* < 0.05; ***P* < 0.01; ****P* < 0.001; #*P* > 0.05 (all *P* values relative to DMSO or PP3 treatment or NC). (**G**), Representative images of IHC staining using the antibody against p-Src (Y419) were shown. Scale bars, 100 μm.

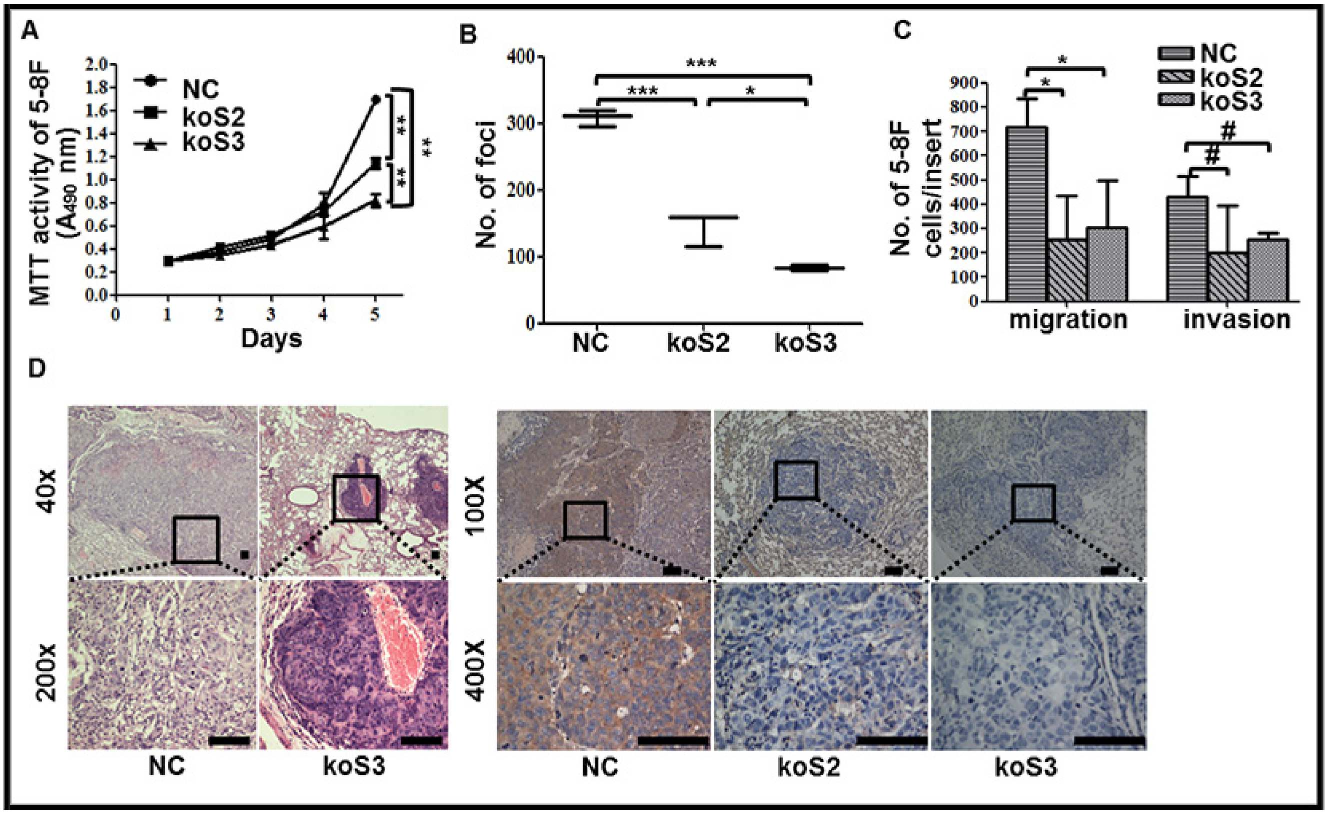

Supplementary Figure S3: The suppression of c-Src activation or expression inhibited malignant capabilities of 5-8F. Knocking out c-Src effectively reduced the viability (**A**) and colony formation ability (**B**) of 5-8F cells. (**C**), Summaries of migrated or invaded 5-8F cells after transfected with CRISPR/Cas9 system containing unique sgRNA targeting c-Src or vector. Abbreviation: koSrc #2 (koS2), koSrc #3 (koS3). (**D**), Representative images of H&E-stained (left panel) and IHC staining against c-Src (right panel) sections derived from lung metastatic nodules after injection with S18-NC or S18-Src. Scale bars, 100 μm.
